# Linking Work Environment to Turnover Intention: The Mediating Role of Moral Distress Among Emergency Nurses

**DOI:** 10.3390/nursrep16060208

**Published:** 2026-06-22

**Authors:** Habib Alrashedi, Omar Almaslamani, Nader Alnomasy, Khalil A. Saleh, Hamdi Lamine, Sameer A. Alkubati

**Affiliations:** 1 Department of Medical Surgical Nursing, College of Nursing, University of Hail, Hail 2440, Saudi Arabia; 2 Emergency Department, King Khalid Hospital, Hail Health Cluster, Hail 55471, Saudi Arabia; 3 Department of Community Health Nursing, College of Nursing, University of Hail, Hail 2440, Saudi Arabia

**Keywords:** moral distress, work environment, emergency, nurses, turnover

## Abstract

**Background/Objectives**: While previous research has explored the effects of moral distress and the work environment separately, there is limited evidence on how these two factors are associated with nurses’ turnover intention. Therefore, in this study, we assessed the mediating role of moral distress in the correlation between nurses’ work environments and turnover intention. **Methods**: This study employed a multicenter cross-sectional design of emergency nurses from April to June 2025. The Measure of Moral Distress—Healthcare Professionals, Practice Environment Scale of the Nursing Work Index (PES-NWI), and Turnover Intention Scale were used to collect data. The mediating effect was analyzed using Hayes’ PROCESS macro (Model 4, Version 4.2) software with the bootstrap technique (5000 repetitions, 95% bias-corrected confidence interval). Statistical significance was set at a threshold of *p* < 0.05. **Results**: Mediation analysis revealed that work environment had a significant negative effect on moral distress (β = −0.251, B = −45.293, 95% CI [−70.376, −20.210], *p* < 0.001). Moral distress significantly increased nurse turnover (β = 0.202, B = 0.008, 95% CI [0.003, 0.012], *p* = 0.003), while the work environment had a significant negative direct effect on turnover (β = −0.391, B = −2.629, 95% CI [−3.507, −1.751], *p* < 0.001). The total effect of work environment on nurse turnover was also significant (β = −0.442, B = −2.970, 95% CI [−3.837, −2.102], *p* < 0.001). Bootstrapping confirmed a significant indirect effect of moral distress (β = −0.051, 95% CI [−0.092, −0.016]), indicating partial mediation. **Conclusions**: This study revealed that nurses’ work environment was significantly associated with turnover intention, both directly and indirectly, through moral distress. Moral distress acted as a statistically significant but modest partial mediator of the association between the work environment and turnover intention, suggesting that it may partially explain this relationship. Strategies by healthcare organizations should be organized to optimize proactive work environments and mitigate moral distress among nurses.

## 1. Introduction

Nurses are the cornerstones of the healthcare system and ensure patient care, safety, and overall hospital efficiency. However, high nurse turnover rates continue to be a global concern, putting a strain on healthcare systems and negatively affecting patient outcomes [1]. One of the key organizational elements linked to nurses’ professional retention and well-being is their work environments. Adequate staffing, leadership support, interprofessional relationships, involvement in decision-making, and access to organizational resources are all components of the nursing work environment [2]. Positive work environments have been linked to nurses’ increased job satisfaction, decreased burnout, improved patient safety, and decreased desire to leave their jobs [3]. Conversely, insufficient staffing, lack of managerial support, and resource limitations may exacerbate occupational stress and encourage nurses to quit their jobs [4]. In particular, emergency nurses serve in extremely challenging clinical settings marked by unexpected patient numbers, trauma exposure, overcrowded environments, critical time-sensitive decision-making, and frequent ethical dilemmas [5]. Compared to many other nursing specializations, emergency nurses are more likely to experience psychological strain, moral distress, burnout, and turnover intention due to these situations [6,7]. Nurses sometimes have to make quick clinical decisions in emergency care settings with few resources and conflicting objectives, which can exacerbate moral dilemmas and emotional weariness [8]. These difficult settings frequently require nurses to offer care under tight time constraints while balancing competing clinical goals. Consequently, emergency nurses regularly face organizational issues that can negatively impact their professional well-being and employment retention.

One of the major factors in challenging work environments is moral distress, a psychological state that arises when emergency nurses feel unable to act in accordance with their ethical views because of institutional limits, conflicting expectations, or systemic impediments [9,10]. Moral distress is especially prevalent in emergency care settings, where nurses often encounter life-threatening situations involving terminal care, insufficient staffing, and ethical quandaries, in which they are obliged to sacrifice patient care owing to hospital policies or limited resources [11]. Unresolved moral distress may eventually lead to emotional exhaustion, burnout, and a strong desire to abandon the field [12,13]. Moral distress has a detrimental effect on nurses’ psychological health and sense of self as a professional. Villagran et al. [14] reported a substantial association between moral distress, burnout, and emotional exhaustion among hospital nurses. Similarly, according to Getahun et al. [15], nurses who deal with moral distress on a regular basis are also more likely to express discontent with their jobs and a strong desire to leave the profession.

Turnover increases healthcare organizations’ recruitment and training costs but also contributes to staff shortages, greater workload for remaining nurses, and lower quality of service [16,17,18]. In addition to moral distress, work environment significantly influences nurses’ job satisfaction and retention. Factors such as leadership support, workplace culture, staffing levels, and ethical climate can either mitigate or exacerbate job-related stress [18,19]. A positive work environment that includes effective communication, adequate resources, and strong managerial support has been linked to lower turnover rates and higher job satisfaction [20]. Conversely, poor working conditions, unsupportive leadership, and ethical conflicts contribute to increased nurse burnout and turnover intention [21]. In emergency departments, where workloads are intense and clinical demands highly unpredictable, supportive work environments are particularly important for maintaining nurses’ well-being and reducing turnover intention [4].

While previous research has explored the effects of moral distress and work environment separately, there is limited evidence on how these factors are associated with turnover intention, specifically within emergency nursing practice. Given the growing demand for emergency departments and global shortage of experienced emergency nurses, understanding these relationships is critical for developing targeted strategies to improve retention, workforce stability, and workplace well-being in emergency care settings.

### Conceptual Framework

This study was guided by the Job Demands–Resources (JD-R) Model and Jameton’s Theory of Moral Distress, which together provide a comprehensive framework for understanding the associations between work environment, moral distress, and turnover intention among emergency nurses [22,23]. According to Jameton, moral distress is the psychological discomfort that arises when a nurse understands what is morally right to do, but feels unable to follow through because of institutional impediments, legal limits, or hierarchical decision-making [23]. According to the JD-R Model, negative work environments marked by high workloads, poor staffing, limited organizational support, and insufficient resources can serve as job demands that contribute to psychological strain and emotional tiredness [22]. These organizational pressures in emergency nursing practice may increase nurses’ exposure to morally problematic situations and diminish their ability to provide care in accordance with professional principles.

Jameton’s Theory of Moral Distress explains that moral discomfort occurs when nurses identify an ethically suitable action but are unable to carry it out because of institutional or structural constraints [24]. The theory suggests that moral distress can lead to emotional exhaustion and burnout as well as decreased job satisfaction. This theory provides a foundation for examining how ethical conflict and systemic constraints contribute to nursing dissatisfaction and turnover. The JD-R Model [22] explains how work-related demands and resources (e.g., moral distress, high workload, understaffing) influence employee stress, engagement, and retention. The model suggests that nurses who experience high job demands and low job resources are more likely to develop burnout and leave their positions, whereas those with supportive work environments are more likely to stay [22].

By combining Jameton’s Moral Distress Theory and the JD-R Model, this study explored the dual impact of moral distress (ethical stressors) and work environment (organizational support) on nurses’ turnover intentions. Understanding these relationships can help develop targeted strategies to mitigate moral distress, enhance workplace conditions, and increase nurses’ retention at work. These findings will help healthcare institutions implement policies to reduce moral distress, enhance workplace conditions, and improve nurses’ retention. Therefore, this study assessed the mediating role of moral distress in the correlation between nurses’ work environments and turnover intention.

## 2. Materials and Methods

### 2.1. Study Design

This study employed a multicenter cross-sectional design.

### 2.2. Population, Sample, and Setting

The target population for this study were emergency nurses working in four primary public hospitals (King Khaled Hospital, Hail General Hospital, King Salman Hospital, and Sharaf Hospital) in the Hail region, an urban administrative region located in northern Saudi Arabia. Eligible participants were registered nurses who (a) were employed in emergency departments, (b) had at least one year of emergency nursing experience, and (c) were willing to participate in the study. Nurses working in administrative positions, interns, nursing students, and those on extended leave during the data-collection period were excluded. Using OpenEpi, Version 3.01 (www.openepi.com), a minimal sample size of 184 emergency nurses was calculated with a 95% confidence level and a 5% margin of error based on a total population size of approximately 350 emergency nurses. To improve representation and consider any non-responses, 200 surveys were sent out. A total of 94.0% of the 188 completed questionnaires that were returned were completed.

### 2.3. Instrument

Moral distress was measured using the Measure of Moral Distress-Healthcare Professionals (MMD-HP), an instrument demonstrated by Epstein et al. [25] to possess strong construct validity and an unstable, high-loading factor structure across diverse clinical settings to evaluate the occurrence and severity of morally upsetting circumstances. The MMD-HP employs a four-factor framework of over 27 items to identify distinct underlying causes of moral distress, which are distributed as follows. Factor 1 (System-Level Root Causes) includes eight elements that evaluate organizational impediments, resource limits, and administrative pressures. Factor 2 (Clinical Root Causes) contains six items that address patient-level concerns, such as administering useless care or witnessing patient pain. Factor 3 (Team-level: Integrity and Vulnerability) contained seven items that reflected intra-team tensions and perceived threats to professional integrity. Finally, Factor 4 (Team-Level: Team/Patient Interactions) includes six elements that address communication failures and ethical issues among the healthcare team, patients, and their families [25,26]. Likert-type responses were used for 27 items, indicating the degree of moral distress (MD) (scoring from none = 0 to considerable distress = 4) and prevalence of each difficulty (scoring from never = 0 to very frequently = 4). Scores for all items (0–432) were added to determine the scale’s total value and the level of distress was multiplied by the frequency of each item. The tool demonstrates strong reliability, with a Cronbach’s alpha of 0.930, indicating excellent psychometric properties [25]. In this study, reliability was confirmed with a Cronbach’s alpha of 0.934.

The Practice Environment Scale of the Nursing Work Index (PES-NWI), a tool internationally recognized for its structural validity and endorsed by the National Quality Forum and developed by Lake [27], was used to measure nursing work environment [27]. The PES-NWI measures hospital environment culture in five main subscales with 31 items as follows: nurse participation in hospital affairs (items 1–9); nursing foundations for quality of care (items 10–19); manager ability, leadership, and support of nurses (items 20–24); staffing and resource adequacy of resources (items 25–28); and Collegial nurse-physician relations (items 29–31) [28]. The 31 items in this measure are all scored on a 4-point Likert scale: a score of one indicates “strongly disagree,” two “disagree,” three “agree,” and four “strongly agree.” The overall score ranged from 31 to 124. The average score is calculated by dividing the total score by 31. According to Lake [27], an average score of 2.5 or higher is considered positive, whereas a score of 2.5 or less is regarded as unfavorable. Cronbach’s alpha for this instrument was 0.940 [29]. In this study, the reliability was assessed using Cronbach’s alpha, which was 0.920.

Turnover intention was assessed using the Turnover Intention Scale developed by Lawler et al. [30]. It consists of three items that measure employees’ turnover intention in their organization (in the near future). Participants scored the items on a 5-point Likert scale ranging from 1 (“extremely disagree”) to 5 (“extremely agree”). The composite score ranged from 3 to 15, with lower scores indicating a lower turnover intention in the current job. The scale has demonstrated acceptable reliability and validity across healthcare and organizational settings, with previous studies reporting satisfactory internal consistency and evidence of construct validity [31]. The scale has demonstrated good reliability with a Cronbach’s alpha value ranging from 0.700 to 0.910 [32,33]. In this study, the reliability was assessed using Cronbach’s alpha, which was 0.907.

To assess the questionnaire’s design viability, readability, and reliability, a pilot study involving 20 emergency nurses was conducted. The results of the pilot study showed that each participant required 15–20 min to complete the questionnaire, which was easy to read and understand.

### 2.4. Data Analysis

The data were coded and cleaned after extraction from online questionnaires. Version 27 of the IBM SPSS Statistics software (IBM Corp., Armonk, NY, USA) was used for the analysis. Descriptive statistics, including frequencies, percentages, means, and standard deviations, were used to summarize the study variables and participant characteristics. The associations between the work environment, moral distress and turnover intention were investigated using Pearson’s correlation coefficient. Data were analyzed using Hayes’ PROCESS macro (Model 4, Version 4.2) implemented in SPSS. A mediation analysis with 5000 bootstrap resamples was conducted to examine whether moral distress mediated the association between the work environment and turnover intention. Demographic variables that demonstrated significant associations with turnover intention in preliminary analyses were entered into the model as covariates. Statistical significance of the indirect association was determined using bias-corrected 95% bootstrap confidence intervals. An indirect association was considered statistically significant when the confidence interval did not include zero.

### 2.5. Data Collection

A convenience sampling strategy was used to recruit the participants. Hospital nursing administrators and emergency department managers were contacted to assist in study dissemination. Eligible nurses were informed of the study aims, eligibility criteria, voluntary nature of participation, and confidentiality assurances before being invited to participate in the study. An online questionnaire was used to collect data from April to June 2025. The survey link was made available electronically through official hospital communication channels and professional nursing organizations. The participants were provided with an electronic informed consent form before they were able to view the questionnaire. Only the nurses who provided informed consent were allowed to participate in the survey. Participants completed the questionnaire on their own and anonymously, taking an average of 15–20 min.

### 2.6. Ethical Considerations

The Institutional Review Board (IRB) of the University of Ha’il (approval number H-2025-708, dated 3 March 2025) and the Ha’il Health Cluster (approval number 2025-33, dated 16 March 2025) approved the study prior to data collection. All procedures involving human participants were conducted in accordance with the ethical standards of the Declaration of Helsinki. The study survey was completed by the participants after they signed an informed consent form. Participants had the option to withdraw at any time. 

## 3. Results

A total of 188 participants were included in this study (Table 1). The mean age was 30.33 ± 6.09 years, and more than half of the sample was male (54.8%). The majority were single (64.4%), without children (68.1%), and predominantly Saudi Arabian (81.4%). With respect to educational level, two-thirds of participants held a bachelor’s degree (67.6%), while one-third had a master’s degree (32.4%).

The mean professional experience was 6.13 ± 5.35 years, with slightly more than half (53.2%) reporting less than five years of work experience. Age, nationality, and years of experience were significantly associated with the patient outcomes. Older participants (≥30 years), non-Saudi nurses, and those with ≥5 years of experience had significantly higher mean scores (*p* = 0.013, *p* = 0.042, and *p* = 0.001, respectively).

Table 2 presents descriptive statistics of the study variables. The mean work environment score (M = 2.84, SD = 0.45, mean percentage score [MPS] = 61.33%) indicated that emergency nurses generally perceived their work environment as moderately favorable. The mean moral distress score (M = 111.14, SD = 82.66, MPS = 25.73%) suggested a relatively low level of moral distress among emergency nurses.

The mean turnover intention score (M = 9.32, SD = 3.07, MPS = 52.67%) reflects a moderate level of intention to leave among emergency nurses.

Table 3 presents the correlations between moral distress, work environment, and nurse turnover. Moral distress was negatively associated with the work environment (r = −0.268, *p* < 0.001), indicating that higher moral distress was associated with less favorable perceptions of the work environment. Moral distress was significantly and positively associated with nurse turnover (r = 0.331, *p* < 0.001), suggesting that greater moral distress was linked to a higher likelihood of turnover.

In addition, the work environment demonstrated a moderate negative association with nurse turnover (r = −0.435, *p* < 0.001), implying that more supportive work environments are associated with reduced turnover intention. For more details on the correlations between the subscales, see Table 3.

**Figure 1:**
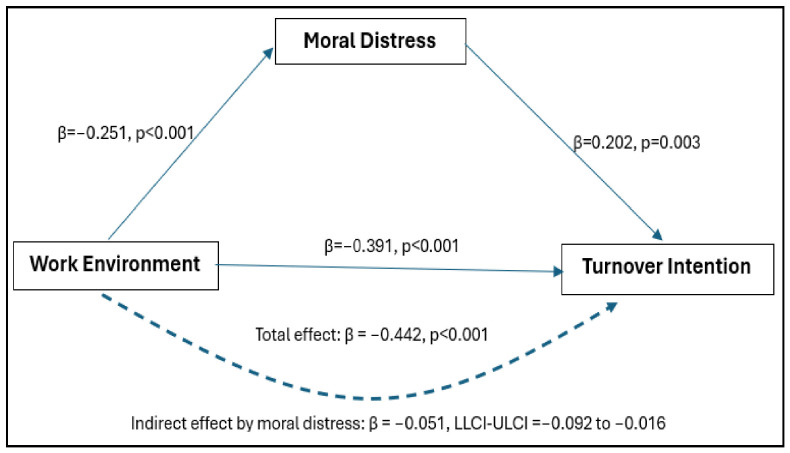
Mediation model examining the association between work environment and turnover intention through moral distress.

Table 4 and Figure 1 present the direct, indirect, and total effects of the work environment on nurses’ turnover intention, with moral distress examined as a mediator among emergency nurses. In the mediator model, work environment had a significant negative effect on moral distress (B = −45.293, β = −0.251, SE = 12.713, t = −3.563, *p* < 0.001), explaining 12.5% of the variance.

In the direct-effects model, moral distress was a significant positive predictor of turnover intention (B = 0.008, β = 0.202, SE = 0.003, t = 3.004, *p* = 0.003). Work environment also showed a significant negative direct effect on turnover intention (B = −2.629, β = −0.391, SE = 0.445, t = −5.907, *p* < 0.001). Among the covariates, experience was a significant positive predictor of nurses’ turnover intention (B = 1.380, β = 0.224, SE = 0.469, t = 2.942, *p* = 0.004), whereas age and nationality were not (*p* = 0.118 and *p* = 0.464, respectively). The model explained 28.1% of the variance in nurses’ turnover intention. The total effect of work environment on nurse turnover intention was statistically significant (B = −2.970, β = −0.442, SE = 0.440, t = −6.753, *p* < 0.001), accounting for 24.5% of the variance.

Bootstrapping analysis indicated a significant indirect effect of the work environment on nurses’ turnover intention through moral distress (β = −0.051, BootSE = 0.019, 95% CI [−0.092, −0.016]), as the confidence interval did not include zero.

## 4. Discussion

This study aimed to investigate the mediating effect of moral distress on the relationship between the work environment and turnover intention among emergency nurses in Hail City, Saudi Arabia. The demographics of our sample, predominantly young, Saudi, and with relatively limited experience (mean experience of 6.13 years), offer important contextual insights. The Saudi health care system has undergone rapid expansion, increasing the number of young Saudi nurses in demanding roles. While the first one to two years post-graduation represent a critical peak period for transitional moral distress, early-career nurses remain highly vulnerable. In our study, more than half of the participants (53.2%) had less than five years of experience. This substantial early-career subgroup remains particularly susceptible to the cumulative stressors of a challenging work environment and moral distress as they consolidate their professional roles in complex clinical settings. Our study adds a crucial layer to this understanding by identifying moral distress as a key mechanism that undermines organizational commitment.

Emergency nursing has unique contextual features that may have amplified the relationships observed in this study. Unlike general medical–surgical units, emergency departments (EDs) are characterized by unpredictable patient flow, high acuity, time-sensitive decision-making, crowding, and frequent interruptions [34]. These conditions create recurrent ethical dilemmas, such as triage decisions that delay care for some patients owing to limited resources or aggressive life support when the prognosis is poor. In this environment, moral distress may arise from daily operational constraints rather than rare events. Similarly, the work environment in EDs, specifically the nurse–physician hierarchy, shift work patterns, and team composition, may have stronger effects on turnover intention than in other settings because ED nurses have fewer opportunities to build patient relationships or control their workflow [8]. Therefore, the mediating role of moral distress identified here should be interpreted within this high-stake emergency context.

Research on nursing in Gulf Cooperation Council (GCC) countries has highlighted unique challenges. Al Omar et al. [35] specifically investigated predictors of turnover intention among Saudi nurses and found that organizational commitment and perceived organizational support were critical factors. Furthermore, a review by el-Gilany and Al-Wehady [36] noted that job satisfaction among Arab nurses is strongly influenced by relationships with supervisors and peers, which are components central to the work environment construct.

Our findings indicate that work environment is a significant predictor of turnover intention among emergency nurses (direct effect = −0.391). Theoretically, these findings can be interpreted using the integrated framework of Jameton’s theory of moral distress and job demands (JD-R) model [22,23]. From this perspective, a challenging work environment may act as a job demand that depletes nurses’ emotional energy. When systemic barriers (e.g., high patient ratios and lack of administrative support for ethical decisions) prevent ethical practice, conditions for moral distress may emerge. Conversely, a positive work environment may act as a job resource that protects against moral distress [10]. This interpretation aligns with the job demands–resources (JD-R) model’s proposition that high demands coupled with low resources lead to burnout and turnover intentions [23,37].

This concept is strongly supported by the work of Lake [27], who developed the Practice Environment Scale of the Nursing Work Index (PES-NWI) and demonstrated that hospitals with better practice environments have significantly lower rates of nurse burnout and job dissatisfaction. Similarly, a systematic review by Woo et al. [37] concluded that poor work environments are one of the strongest predictors of nurses’ turnover intention globally. Our results align with the claims of the JD-R model that high demands coupled with low resources lead to negative outcomes such as burnout and turnover intention [24]. The moderately favorable mean score (2.84 ± 0.45) in our sample suggests that there is room for improvement; even some enhancements in leadership support, staffing levels, and resource availability could yield substantial benefits for retention.

Furthermore, the positive correlation between moral distress and turnover intention (r = 0.331, *p* < 0.001) and the significant direct path in the mediation model (effect = 0.202) suggest that moral distress is strongly associated with nurses’ desire to leave. For instance, a study by Epstein and Hamric [38] describes the “crescendo effect” of moral distress, where unresolved ethical conflicts accumulate, leading to emotional exhaustion and a fundamental desire to escape the situation causing distress, often by leaving the job or the profession altogether. Similarly, a recent study in Ethiopia by Getahun et al. [15] found that moral distress was a significant predictor of nurses’ turnover intention. 

Maunder et al. [39] conducted a longitudinal analysis of moral distress and found it to be a more potent predictor of intention to leave than the general burnout measures. They contended that moral distress targets the desire to deliver quality care, which is the main reason that nurses enter the field. The psychological contract between the organization and the nurse is shattered when this basic goal is consistently not met, resulting in disengagement and departure. Furthermore, Singhal and Chukkali [40] highlighted that moral distress in India is significantly associated with feelings of guilt and shame, which are powerful emotional drivers seeking a way out of a distressing situation.

The mean score for the work environment in our sample suggests a moderately favorable perception. However, the significant correlation with turnover intention indicates that, even within this range, variations in environmental quality have a substantial impact. Factors such as nurse-to-patient ratio, leadership support, and interdisciplinary collaboration are critical. The evidence from McHugh et al. [41] is particularly relevant. Their multi-country study demonstrated that better nurse-to-patient ratios, a key component of the work environment, were directly associated with lower burnout and improved retention.

This finding adds further evidence to the existing literature and aligns with the call of Ulrich et al. [42] to investigate the multifaceted nature of the nurses’ work environment, including its ethical dimensions. A toxic or unsupportive environment creates “institutional constraints,” which Jameton identifies as the root of moral distress. For example, understaffing (a work environment flaw) forces nurses to ration care, leading to the ethical dilemma of knowing what quality care should be, but being unable to provide it. This unresolved distress manifests as emotional exhaustion and disengagement that precede turnover intention, as described in the JD-R model.

Our results were supported by both qualitative and quantitative evidence. Bondjers [9] argues that a “protective work environment” is essential for mitigating moral distress, especially during crises. Similarly, a scoping review by Ahmad [43] identified factors such as inadequate communication, a lack of ethical support, and perceived institutional injustice as key contributors to moral distress, all of which are elements of the work environment. Our study quantitatively bridges these concepts, showing that the effect of these environmental deficiencies on turnover intention is partially mediated by the experience of moral distress.

### 4.1. Strengths and Limitations

This study had several limitations. First, although the mediation study revealed that moral distress could be a psychological link between the work environment and turnover intention, the observed relationships were statistical associations rather than causal effects. Therefore, the results of this study should be interpreted with caution because the cross-sectional design does not allow for conclusions on temporal ordering or causality. Longitudinal research is required to assess whether unpleasant work settings contribute to increased moral distress over time, and whether persistent moral distress increases nurses’ turnover intention. Second, all variables were measured using self-report questionnaires completed by the same participant at a single time point. This approach may have introduced recall, social desirability, and common method biases, potentially inflating the observed associations. The use of time-lagged designs or multi-source assessments (e.g., supervisor-reported outcomes and observer-rated work environments) would help address this limitation.

Third, this study treated moral distress and work environment as unidimensional summary scores. Further investigation of the variable subdomains may help identify which aspects of the work environment and moral distress are most strongly associated with turnover intention.

Fourth, mediation analysis was carried out using the observed composite scores obtained from the study instruments. Although this method is commonly used, it does not account for measurement errors, which may affect the estimated relationships between the variables. Longitudinal designs should consider using latent-variable modeling methodologies, such as structural equation modeling, to account for measurement errors and produce more precise estimates of indirect connections.

Fifth, the sample was drawn from a single city in Saudi Arabia using convenience sampling, and most participants were young, single, or Saudi Arabian. The findings may not be generalizable to other nursing specialties (e.g., intensive care and medical–surgical), other regions within Saudi Arabia, or healthcare systems with different cultural, organizational, or staffing models.

Despite these limitations, this study had several strengths. It is one of the first in the region to explicitly test a mediation model linking work environment and turnover through moral distress. The use of validated instruments and robust statistical methods, including bootstrapping for mediation analysis, strengthened the validity of the findings. Furthermore, the focus on emergency nurses, a population that is highly vulnerable to both moral distress and burnout, adds to the practical significance of the results.

### 4.2. Implications for Policy and Practice

The implications of these findings are substantial, and call for a multi-faceted approach by nursing leaders and policymakers. Proactive Work Environment Optimization: Healthcare organizations must move beyond reactive retention strategies. Investments must be made in evidence-based practices known to improve the work environment, such as implementing primary nursing models, ensuring nurse involvement in policymaking committees, and guaranteeing safe nurse-to-patient ratios using validated acuity tools.

Structural Empowerment to Mitigate Moral Distress: Creating a “moral community” is essential. This involves establishing structured ethics support systems such as regular ethics rounds or rapid-response ethics consultation services. These structures empower nurses by giving them a voice and pathway to address ethical dilemmas, thereby reducing the isolation and helplessness that characterize moral distress.

Leadership Development: Nurse managers play a pivotal role. Leadership training should focus on developing “ethical leadership” skills—the ability to recognize signs of moral distress in staff, create a climate of psychological safety where ethical concerns can be raised, and effectively advocate for resources and policy changes on behalf of the nursing team.

## 5. Conclusions

In summary, this study provides evidence that work environment plays a significant role in determining retention among emergency nurses, both directly and indirectly, through moral distress. These findings emphasize the importance of developing organizational strategies tailored specifically to emergency-care settings. Interventions focusing on staffing support, ethical consultation services, leadership responsiveness, and psychological support mechanisms may help reduce moral distress and strengthen retention among emergency nurses. Moral distress may partially explain the association between work environment and turnover intention among emergency nurses. However, causal inferences could not be made because of the cross-sectional design. Future longitudinal research is required to examine these relationships and their temporal sequences further.

## Figures and Tables

**Table 1:** Sociodemographic characteristics of participants (N = 188).

Variables	Categories	n	%	Mean ± SD	t/f (*p*-Value)
Age (years)	30.33 ± 6.09				
	<30	109	58.0	8.85 ± 3.03	−2.499 (0.013)
	≥30	79	42.0	9.97 ± 3.04	
Gender					
	Male	103	54.8	8.99 ± 3.21	−1.645 (0.102)
	Female	85	45.2	9.72 ± 2.87	
Marital status					
	Single	121	64.4	9.01 ± 2.98	2.451 (0.089)
	Married	55	29.3	10.09 ± 3.31	
	Other	12	6.4	8.91 ± 2.46	
Nationality					
	Saudi	153	81.4	9.11 ± 3.11	−2.080 (0.042)
	Non-Saudi	35	18.6	10.22 ± 2.78	
Children					
	No	128	68.1	9.06 ± 2.88	−1.712 (0.088)
	Yes	60	31.9	9.88 ± 3.41	
Educational level					
	Bachelor	127	67.6	9.17 ± 3.06	−0.972 (0.333)
	Mateer	61	32.4	9.63 ± 3.10	
Experience (years)	6.13 ± 5.35				
	<5	100	53.2	8.65 ± 3.11	−3.284 (0.001)
	≥5	88	46.8	10.09 ± 2.86	

t: Independent *t*-test, f: ANOVA test.

**Table 2:** Statistical description of variables (N = 188).

Variables	Range	Minimum	Maximum	Mean ± SD	MPS
Work environment	2.50	1.50	4.00	2.84 ± 0.45	61.33
Moral distress	432.00	0.00	432.00	111.14 ± 82.66	25.73
Turnover intention	12.00	3.00	15.00	9.32 ± 3.07	52.67

MPS: Mean Percentage Score.

**Table 3:** Correlation between study variables.

Variables	1	2	3	4	5	6	7	8	9	10	11	12
1. Nurse Participation in Hospital Affairs	1											
2. Nursing Foundations for Quality of Care	0.814 **	1										
3. Nurse Manager Ability, Leadership, and Support of Nurses	0.412 **	0.683 **	1									
4. Staffing and Resource Adequacy	0.396 **	0.706 **	0.913 **	1								
5. Collegial Nurse-Physician Relations	0.492 **	0.703 **	0.932 **	0.922 **	1							
6. Work Environment (Total)	0.770 **	0.935 **	0.869 **	0.864 **	0.888 **	1						
7. System-Level Root Causes	−0.265 **	−0.264 **	−0.162 *	−0.128	−0.166 *	−0.242 **	1					
8. Clinical Root Causes	−0.165 *	−0.191 *	−0.115	−0.125	−0.127	−0.173 *	0.914 **	1				
9. Team-Level: Integrity and Vulnerability	−0.332 **	−0.343 **	−0.207 **	−0.166 *	−0.209 **	−0.311 **	0.908 **	0.734 **	1			
10. Team-Level: Team/Patient Interactions	−0.329 **	−0.346 **	−0.206 **	−0.181 *	−0.208 **	−0.313 **	0.921 **	0.792 **	0.973 **	1		
11. Moral Distress (Total)	−0.284 **	−0.294 **	−0.179 *	−0.150 *	−0.183 *	−0.268 **	0.988 **	0.906 **	0.946 **	0.961 **	1	
12. Turnover intention	−0.413 **	−0.452 **	−0.322 **	−0.293 **	−0.316 **	−0.435 **	0.326 **	0.243 **	0.362 **	0.336 **	0.331 **	1

** Correlation is significant at the 0.01 level (2-tailed). * Correlation is significant at the 0.05 level (2-tailed).

**Table 4:** Direct, Indirect, and Total Effects of Work Environment on Nurse Turnover intention with Moral Distress Mediation.

Path	B	β	SE	t	95% CI for B	*p*-Value
Work Environment → Moral Distress	−45.293	−0.251	12.713	−3.563	70.376 to −20.210	<0.001
Model Summary R^2^ = 0.125, F = 6.535, *p* < 0.001						
Direct effect						
Moral Distress → Nurse Turnover intention	0.008	0.202	0.003	3.004	0.003 to 0.012	0.003
Work Environment → Nurse Turnover intention	−2.629	−0.391	0.445	−5.907	−3.507 to −1.751	<0.001
Age (Covariate)	−0.062	−0.123	0.040	−1.569	−0.140 to 0.016	0.118
Nationality (Covariate)	0.419	0.053	0.571	0.734	−0.708 to 1.545	0.464
Experience (Covariate)	1.380	0.224	0.469	2.942	0.455 to 2.306	0.004
Model Summary R^2^ = 0.281, F = 14.236, *p* < 0.001						
Total effect						
Work Environment → Nurse Turnover intention	−2.970	−0.442	0.440	−6.753	−3.837 to −2.102	<0.001
Model Summary R^2^ = 0.245, F = 14.886, *p* < 0.001						
Indirect effect			β	BootSE	BootLLCI	BootULCI
Work Environment → Moral Distress → Nurse Turnover intention			−0.051	0.019	−0.092	−0.016

B: unstandardized coefficient; β: standardized coefficient; SE: standard error; t: t-value; CI: confidence interval; LLCI: lower limit of confidence interval; ULCI: upper limit of confidence interval; *p*: probability value; R^2^: coefficient of determination; F: F-statistic; BootSE: bootstrap standard error.

## Data Availability

The data presented in this study are available upon request from the corresponding author due to privacy or ethical restrictions.

## Public Involvement Statement

No public involvement in any aspect of this research.

## Guidelines and Standards Statement

This manuscript was prepared in accordance with the STROBE (Strengthening the Reporting of Observational Studies in Epidemiology) guidelines for cross-sectional studies.

## Abbreviations

The following abbreviations are used in this manuscript:

JD-R	Job Demands-Resources
MMD-HP	Measure of Moral Distress for Healthcare Professionals
PES-NWI	The Practice Environment Scale of the Nursing Work Index
